# Transcriptional responses to nitric oxide are widescale and source-dependent

**DOI:** 10.1016/j.jbc.2025.110476

**Published:** 2025-07-11

**Authors:** Joseph C. Schindler, Puneet Seth, Richard T. Premont, Jonathan S. Stamler

**Affiliations:** 1 Institute for Transformative Molecular Medicine, Department of Medicine, Case Western Reserve University School of Medicine, Cleveland, Ohio, USA; 2 Department of Biochemistry, Case Western Reserve University School of Medicine, Cleveland, Ohio, USA; 3 Harrington Discovery Institute, University Hospitals Cleveland Medical Center, Cleveland, Ohio, USA

**Keywords:** nitric oxide, S-nitrosylation, nitric oxide synthase, gene transcription, transcriptome

## Abstract

The transcriptomic effects of nitric oxide (NO) have been widely studied across phylogeny. However, while gene expression is canonically altered by NO, general principles have not emerged. Here, we characterize genetic regulation within a single cell type after exposure to NO derived from endogenous or synthetic donor compounds or produced by 3 different NO synthase (NOS) isoforms under basal and activated conditions. Using RNAseq, we uncover distinct, source-dependent effects of NO on as many as ∼10,000 genes mediated largely by S-nitrosylation. NOS enzymes and NO donors each generated unique transcriptional responses. Our data reveal non-overlapping transcriptional responses to NO that are likely mediated by distinct effectors and enzymes and highlight that NO-treated cell systems may undergo a dramatic and widespread transcriptional response.

Nitric oxide (NO) is a ubiquitous redox signaling molecule with diverse roles in health and disease (1). While early views highlighted the propensity for NO to signal *via* cGMP/protein kinase G (1) (*i*.*e*., indirectly *via* phosphorylation), this perspective overlooked NO’s redox function and has been superseded by direct enzyme-mediated signaling *via* the formation of S-nitrosothiols (SNOs; termed S-nitrosylation) (2). To this end, thousands of proteins have been reported to become S-nitrosylated under various cellular conditions (3) and thereby regulate myriad cellular systems, including receptor tyrosine kinase (4) and G protein-coupled receptor functions (5), ligand-gated ion channels (6), and gene expression (7). Aberrant SNO signaling is also widely implicated in numerous pathologies across organ systems, including the brain (8), kidney (9), heart (5), and lung (10).

Redox signaling by NO is carried out by 4 classes of enzymes (11). First, NO is produced by 3 isoforms of nitric oxide synthase (NOS): NOS1 and NOS3 are activated by calcium (12) whereas NOS2 is transcriptionally regulated (13). NO then undergoes oxidation by transition metals within a SNO synthase to be covalently fixed on thiols, forming SNO-proteins and low molecular weight SNOs (2, 11, 14). The SNO functional group is subsequently transferred between cellular thiols *via* transnitrosylase enzymes (writers) like SCAN (4), leading to its placement on specific protein targets to alter function. Signaling is terminated by denitrosylase enzymes (erasers) such as SNO-CoA reductase 2 (SCoR2) (15), analogous to phosphatases and deacetylases (16). Thus, signaling *via* NO is highly specific, involving multiple classes of enzymes acting in concert and requiring redox and carrier intermediates to exert precise effects.

Regulation of gene expression is high among NO’s canonical functions (17, 18), including direct effects of S-nitrosylation on transcriptional regulators (19, 20), epigenetic histone remodelers (21, 22), miRNA regulators (22, 23), and nuclear trafficking machinery (24). NO’s effects on transcription act across virtually all cell types, are shared between eukaryotes and prokaryotes, and are well demonstrated in plants (25), fungi (26), bacteria (27, 28) and humans (7).

While many studies have demonstrated NO’s effect on defined gene targets, few principles have emerged. One limitation of these studies has been almost complete reliance on chemical NO donors as proxies for endogenous production of NO by NOS. However, recent data have suggested that NO donors depend on writer enzymes to mediate their effects rather than acting *via* direct chemical reaction (29). Moreover, steady-state SNO levels are controlled primarily by denitrosylases (2, 30), not NOSs (9, 29). Thus, chemical and enzymatic sources of NO may represent complementary components of the NO response. There is therefore a need for a more systematic approach to studying NO signaling.

A number of factors have been shown to influence the genetic response to NO, including dose- and time-dependent (31) effects. Somewhat surprisingly, the principal effect of NO is seen within a narrow concentration range (32), indicating that dose alone cannot account for the results. Furthermore, different cell lines have entirely different responses to the same NO donor compound (33), limiting general conclusions. Here we use multiple chemical and enzymatic approaches to NO generation in a single model cell, which can be globally assessed through transcriptomic fingerprints, to overcome these limitations. Our results reveal that the molecular source of NO—not its presence or concentration—drives unique transcriptional output at a large scale.

## Results

### NOS isoforms direct unique responses to NO

**Figure 1:**
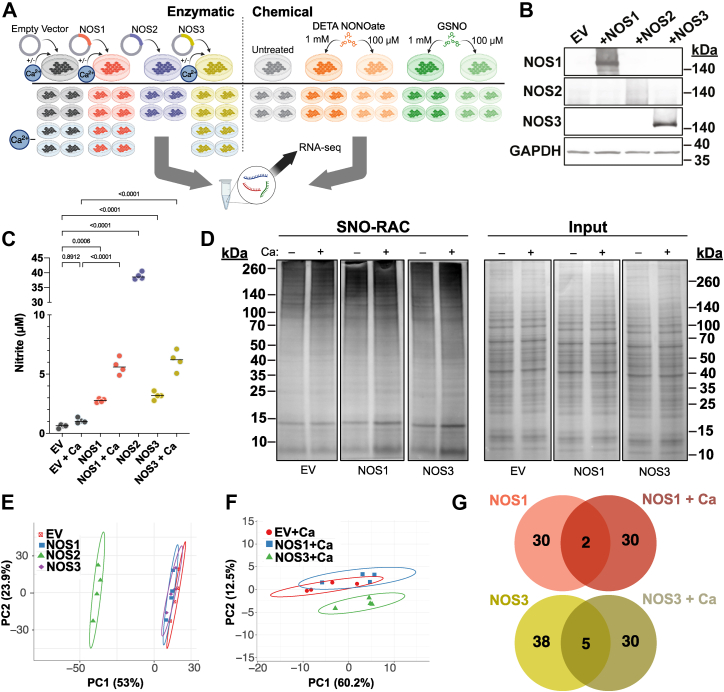
**Distinct NOS activities, protein S-nitrosylation and transcriptional responses**. *A*, schematic of the experimental design. Created with BioRender.com. *B*, immunoblot confirming expression of each NOS isoform. *C*, nitrite in cell culture medium of transfected cells measured by Griess assay. *p*-values are for pairwise comparisons *via* ANOVA using Sidak multiple test correction. *D*, representative (*n* = 3) silver-stained SNO-RAC after calcium activation of NOS1 and NOS3 relative to Coomassie-stained inputs. *E–F*, PCA of *n* = 4 replicates from enzymatic treatments at baseline (*E*) or with Ca (*F*), with EV controls. Ovals = 90% confidence intervals. *G*, comparison of significantly altered genes between NOS1 and NOS3 at baseline and after Ca treatment.

To determine whether NO derived from different sources has similar or distinct effects on gene expression, we expressed NOS1, 2, 3, or an empty vector (EV) control in HEK293 cells. We treated NOS1 and NOS3 cells with A23187 calcium ionophore (Ca) to stimulate NOS activity (plus EV+Ca control); NOS2 is constitutively active (Fig. 1*A*). After confirming NOS expression (Fig. 1*B*), we assayed culture media for the presence of nitrite, a byproduct of NO production (34). Nitrite concentrations were highest when NOS2 was overexpressed, in accordance with its high NO output (35), and more modestly elevated for NOS1 and NOS3. Ca treatment increased nitrite production by NOS1 and NOS3, but not EV (Fig. 1*C*). Using SNO-RAC to capture SNO-proteins, we demonstrated that Ca induced S-nitrosylation across the proteome in NOS1 and NOS3 cells but had no effect on the SNO-proteome of EV cells (Fig. 1*D*).

**Figure 2:**
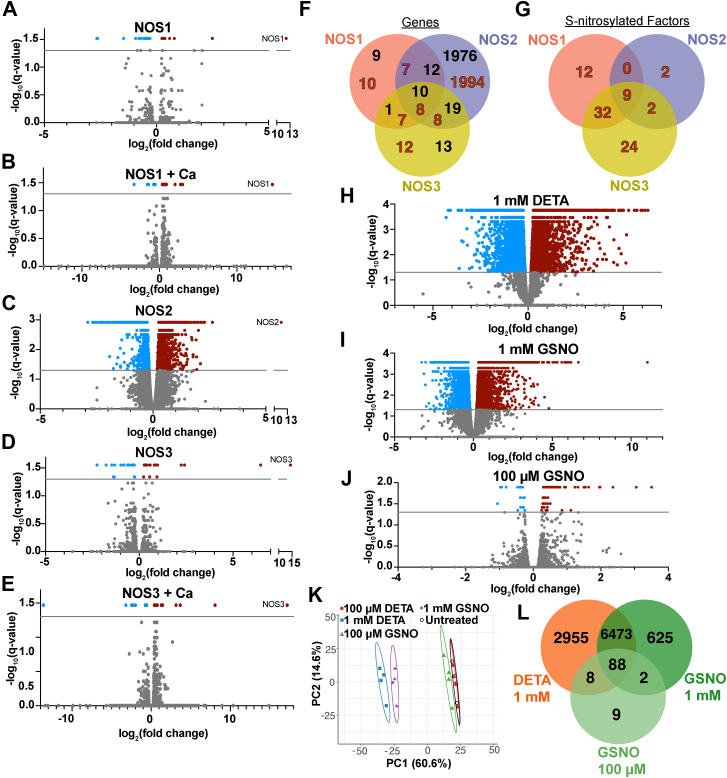
**Differentially expressed genes after treatment with various NO sources**. *A–E*, volcano plots indicating differentially expressed genes in cells of indicated enzymatic treatments, each compared to respective EV controls. *Red* = upregulation, *blue* = downregulation above the significance threshold (*line*); non-significant transcripts are in *gray*. *F*, comparison of the differentially expressed genes in cells expressing each NOS enzyme. *Black* = NOS enzymes at baseline, *red* = NOS1+Ca and NOS3+Ca. *G*, comparison of numbers of transcript-regulating factors S-nitrosylated by each NOS enzyme (from a previously published mass spectrometry dataset (41) under identical Ca conditions). *H–J*, volcano plots as in (*A–E*) for chemical donor treatments relative to untreated controls. *K*, PCA of *n* = 4 replicates from conditions in (*H–J*) plus the untreated control and the 100 μM DETA treatment. Ovals = 90% confidence intervals. *L*, comparison of the numbers of differentially expressed genes in cells treated with the 3 conditions that led to transcriptomic changes.

Next, total RNA was purified for sequencing (Fig. 1*A*) and differential expression analysis was performed to identify significantly altered genes (relative to control) for each NOS condition. Overall, transcriptional changes seemingly correlated with nitrite levels, so NOS2 substantially reshaped the transcriptome while NOS1 and NOS3 exerted modest effects over EV, as observed in principal component analysis (PCA) (Fig. 1*E*). However, while NOS1+Ca and NOS3+Ca (Fig. 1*F*) showed higher nitrite than under basal conditions (Fig. 1*C*), the absolute numbers of differentially regulated genes with and without Ca activation were essentially identical. Instead, the gene targets of NOS1 and NOS3 enzymes changed almost completely with Ca (Fig. 1*G*), suggesting these 2 physiological states signal through distinct pathways to alter transcriptional responses. Specifically, we uncovered 32 genes whose expression changed significantly with NOS1 and 32 genes with NOS1+Ca (but these genes were largely distinct); 2017 genes under the regulation of NOS2; and 43 genes regulated by NOS3 and 35 by NOS3+Ca (again largely distinct) (Fig. 2, *A–E*). Thus, effects of NOS on gene transcription cannot be linked simply to amount of NO.

Our data raise the idea that the enzymatic source of NO plays a role independent of NO concentration. Indeed, while some genes may be affected by NO from any NOS, most genes were uniquely regulated by different enzymes, regardless of Ca or amount of NO (Fig. 2*F*). This finding is in accord with previous work showing that SNO-proteomes are unique products of protein interactomes built around individual NOS isoforms (36). To support this mechanistically, we analyzed previously published mass spectrometry data of proteins S-nitrosylated downstream of each NOS under identical conditions (36). Cross-referencing sets of transcription factors (37) and epigenetic regulators (chromatin remodelers, histone modifiers, chaperones, and direct nucleic acid modifiers) (38) with NOS-specific SNO-proteomes generated sets of differentially nitrosylated candidates that could explain the differential effects on gene expression we have observed (Fig. 2*G*).

### Large-scale transcriptomic responses to NO donors

To further explore the specificity of NO on gene expression suggested by these NOS experiments, we treated cultured cells with chemical donors at high (1 mM) or low (100 μM) concentrations (Fig. 1*A*). Specifically, we chose DETA NONOate, a synthetic NO donor compound that slowly releases NO at low nanomolar concentrations with a half-life of 20 h at 37° C; this NO must be redox activated to form SNOs. We also used S-nitrosoglutathione (GSNO), an endogenous low molecular weight SNO that rapidly transfers its NO group to protein targets (2); GSNO has been previously used at 100 μM for studying transcription (33).

In agreement with the apparent dose dependency in the NOS data, both compounds exerted widespread effects on expression of many thousands of genes at the higher dose, while the lower dose conditions induced far fewer changes, and in the case of 100 μM DETA, did not significantly affect gene expression at all (either due to very low NO concentrations or because of active denitrosylation) (Fig. 2, *H–K*). Interestingly, most gene targets of high-dose DETA and GSNO were shared (Fig. 2*L*), though each had unique targets. Also noteworthy is that almost all targets of low-dose GSNO were also targets of high-dose conditions. Thus, unlike NOSs, which had largely non-overlapping effects, donors (with distinct structures, cellular uptakes, NO levels, and NO chemistries) elicited some similar effects. Altogether, these data suggest that concentration alone cannot account for transcriptional responses.

### Source-dependent genetic targets of NO mediate specific responses

**Figure 3:**
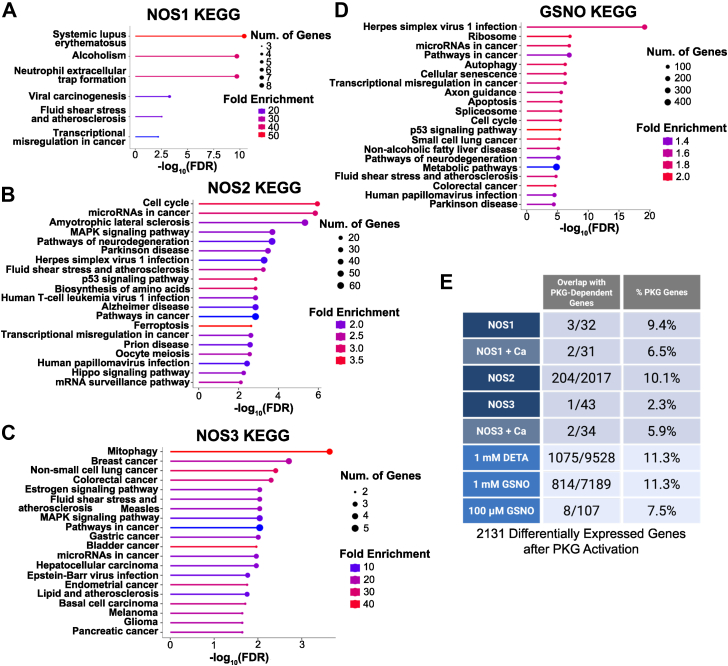
**Ontology analyses of transcriptional targets of NO from distinct sources**. *A–D*, enrichment of KEGG pathways for indicated conditions (NOS1 and NOS3 at baseline, GSNO at 1 mM). *E*, NO-dependent genes from all NOS and donor datasets were compared to a previously published RNAseq dataset of transcripts altered by treatment with a direct PKG activator (56); the number of genes in each set overlapping with PKG-dependent genes is shown as a fraction and percentage.

We next used KEGG to analyze genes and pathways regulated by alternative sources of NO. Data on NOSs largely conformed to the known physiological roles of each isoform (Fig. 3, *A–C*), suggesting that each mediates targeted transcriptional responses. Specifically, pathways regulated by NOS1 (neuronal NOS) (39) were enriched for genes in atherosclerosis (40, 41) and neutrophil extracellular traps (42, 43); genes regulated by NOS2 (inducible NOS) include cell cycle control (44) and inflammatory processes, *i*.*e*., hallmarks of the interferon response that induces NOS2 (45); and genes regulated by NOS3 (endothelial NOS) play a role in cancer pathogenesis by promoting angiogenesis (46), atherosclerosis (47, 48), and estrogen-mediated vasoactive signaling (49, 50).

The 2 chemical NO donors showed largely overlapping targets (Fig. 3*D*; only GSNO is shown due to a few differences with DETA). Further, whereas NO from NOSs was associated with unique cellular responses, NO from chemical donors was less selective. As further evidence for differing patterns, basal datasets combined identified just 3 genes with consistently altered expression regardless of NO source or concentration. Moreover, while all 3 genes exhibited large changes in expression (∼10-fold), directionality depended on the NO source. For example, the transcription factor *ATF3* is markedly upregulated by NOS2, 1 mM GSNO, and 1 mM DETA treatments but markedly downregulated by NOS1, NOS3, and 100 μM GSNO. We conclude from the magnitude of changes and the identities of targets that NO donors do not replicate NOSs, and each enzyme and donor may have unique, concentration-dependent effects.

### Transcriptional targets of NO donors and NOSs are substantially unique

**Figure 4:**
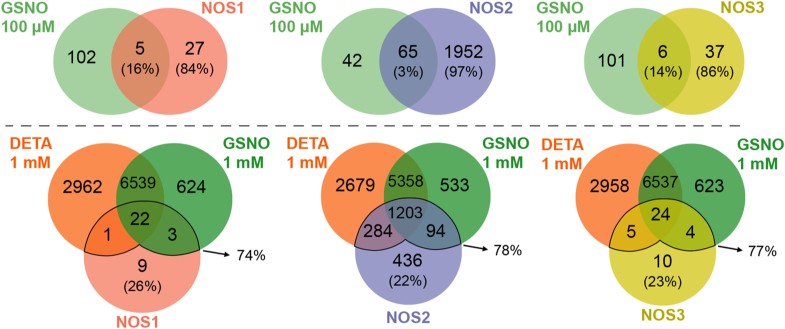
**Comparisons of transcriptional targets of NOS isoforms and chemical donors**. Genetic targets of each NOS isoform (at *baseline*) compared to targets of 100 μM GSNO (*top* cluster), and with 1 mM DETA and 1 mM GSNO (*bottom* cluster). Percentages of the targets of NOS are displayed for genes commonly affected by the enzyme and the chemical donor(s) as well as uniquely affected by the enzyme.

We next directly compared the targets of endogenous (GSNO) and synthetic (DETA) donors to those of the 3 NOS enzymes (Fig. 4). Genetic targets of 100 μM GSNO showed very little overlap with any NOS isoform, suggesting that GSNO activity is distinct from NOS activity. By contrast, 1 mM DETA or GSNO shared many targets with each NOS isoform, but also included thousands of additional genes, highlighting the specificity of NOS enzymes *versus* the promiscuity of chemical donors. We conclude that NO donors have different targets than NOS-derived NO, with lower doses failing to replicate NOS effects altogether and higher doses acting less specifically. Thus, essentially all genetic regulation by NO is tethered to the source of its production.

### NO signals largely through S-nitrosylation

Finally, we sought to determine how many NO-regulated genes are regulated by the traditional cGMP-PKG pathway (1), compared to S-nitrosylation. We utilized a previously generated RNAseq dataset of differentially expressed genes after treatment with the PKG activator 8-Br-cGMP (51). Comparing these PKG-dependent genes with the NO-dependent genes identified in our RNAseq showed that only ∼10% or fewer—irrespective of enzymatic or chemical source or NO concentration—were also in the PKG dataset (which contained 2131 genes) (Fig. 3*E*). Moreover, effects of PKG may be mediated in part by S-nitrosylation (52, 53). Altogether, a large majority of gene expression changes by NO (∼90%) appear to be mediated by S-nitrosylation rather than cGMP activation.

## Discussion

In this study, we deconstruct genetic responses to NO derived from different sources using a single cell type to allow for direct comparisons. Surprisingly, we reveal unique responses to each condition, *i*.*e*., to different NOS isoforms, different NO donors, enzymes vs. chemicals, and basal vs. activated NOS. Transcriptional effects of NO are not simply dose-dependent, as one might expect, but rather non-overlapping, emphasizing distinct targets and machinery (2, 4, 9, 11, 29) for protein S-nitrosylation. Altogether, thousands of genes may be transcribed in response to NO, broadly changing the cellular landscape. Such widescale effect of NO on the transcriptome has not been previously recognized. Different NO treatments may thus result in distinct cellular proteomes, confounding interpretations of data.

NO signaling requires a series of enzymatic steps from the production of NO to its conversion to SNO and subsequent transfer of SNO onto protein effectors (2, 4, 28, 29), and physiological systems contain numerous enzymes that subserve NO-responsive transduction. Denitrosylases then restore homeostasis and establish the steady state condition (2, 9, 30). In isolation, neither NO, nor compounds that release it, nor enzymes that produce it are sufficient to recapitulate this physiology. Together, however, the different sources of NO may reconstitute canonical responses. This is well illustrated in the transcriptional activity of NO donors and NOSs. NO donors (*e*.*g*., NONOates, GSNO) have long been used based on the assumption they replicate generic NOS activity, while basal vs. activated NOS assumes a linear response. But in fact, NO donors do not recapitulate NOSs, and the effects of basal and stimulated NOS activity are almost entirely non-overlapping. This can be understood by appreciating that donors may better phenocopy the activity of transnitrosylases than NOSs (4, 29) and that basal SNOs are primarily under the control of denitrosylases (15, 30). That is, steady-state SNO levels are not determined by NOSs. We further demonstrate that the transcriptional response to donor compounds approached 10,000 genes, implying that treatments commonly employed to target individual proteins may instead reshape the entire transcriptome. Our data suggest broad-scale changes to cells treated with NO that raise important caveats with the NO literature.

Our work supports a dose-dependent effect of NO on gene expression (32, 54, 55) (*e*.*g*., NOS2 > NOS1, 3, and 1 mM > 100 μM), yet the scope of transcriptional responses cannot be explained by the amount of NO alone. While NO donors were used at identical concentrations, they generate very different amounts of bioactive NO. DETA generates sustained low concentrations of NO over many hours (56) while SNOs like GSNO act primarily as direct nitrosylating agents with short half-lives (57). By the same token, stimulation of NOS activity with Ca increased the amount of NO, but not the number of genes transcribed. Instead, the identity of target genes changed. Similarly, NOS2 generated more NO and a greater transcriptional response than NOS1 or NOS3, but its effect was non-overlapping with low or high amounts of NO generated from alternative sources. Our data support the idea that the effects of NOSs are more dependent on the company they keep (within isoform-specific interactomes) (36) than the concentration of NO they generate, and that these interactomes may be significantly altered by physiological signal transduction, such as binding of calcium, which introduces a conformational change in NOS enzymes (58). Furthermore, previously published mass spectrometry data identify different transcription factors S-nitrosylated by different NOSs, likely explaining the observed effects (36). More generally, whereas increasing NO roughly correlates with more transcriptional events, it does not necessarily correlate with the amounts of S-nitrosylated proteins. This can be understood by appreciating that S-nitrosylation events (36) are enzymatically mediated (involving many different enzymes) (2, 4, 9, 28, 29). Altogether, our data highlight the concept that different machinery operates under different cellular conditions to transduce the redox-mediated response to NO.

## Experimental procedures

### Plasmids

cDNAs for the expression of human NOS1, NOS2, or NOS3 were cloned into the pcDNA3.1 Zeo(+) mammalian expression vector backbone. The parent vector was used as the EV control.

### Cell culture and transfection

HEK293 cells were cultured in DMEM+10% Fetal Bovine Serum with 1% Antibiotic-Antimycotic (Gibco). Prior to transfection, cells received fresh media, additionally supplemented with 1 mM L-arginine. Six-well plates were transfected using Lipofectamine 3000 Transfection Reagent (Invitrogen) with 2.5 μg of DNA per well.

### Activation of NOS1 and NOS3

To activate NOS1 and NOS3 (and EV control) above baseline activity, after transfection with expression plasmids, cells were treated with 5 μM calcium ionophore A23187 (Sigma-Aldrich) for 8h prior to harvest.

### Treatment with chemical donors- DETA NONOate and GSNO

Prior to treatment, HEK293 cells received fresh media with 1 mM L-arginine. DETA NONOate (Cayman Chemical) was dissolved in 0.01 N NaOH, serially diluted, and added to culture medium at final concentrations of either 1 mM or 100 μM.

GSNO was generated by reacting equimolar reduced L-glutathione (GSH; MilliporeSigma) with NaNO_2_ to generate GSNO, which was immediately serially diluted and added to culture medium at final concentrations of either 1 mM or 100 μM.

### Extraction and sequencing of RNA

After 24h of transfection or chemical treatment, RNA was purified from cultured cells by direct lysis in TRIzol Reagent (Invitrogen) and submitted to the CWRU Genomics Core for QC, library preparation, and paired-end, 150 nt sequencing with ∼42 million reads per library on the NovaSeq X platform (Illumina). Raw sequencing data are available at GEO (GSE295064).

Sequence reads were subjected to QC filtering using TrimGalore! v0.4.2 (Babraham Bioinformatics), a wrapper script for FastQC and cutadapt. Reads passing QC and with adapter sequences trimmed were aligned to the human GRCh38 reference genome using STAR aligner v2.7.9 (59) with GENCODE annotations and analyzed for differential expression using Cufflinks v2.2.1 (60). Expression values were reported as fragment counts per kilobase of exon per million fragments mapped (FPKM). Differentially expressed genes were identified using a cutoff of FDR-adjusted q-value<0.05. Transcripts with both significantly decreased and increased expression were used for comparisons; KEGG analyses *via* ShinyGo v0.80 (61).

### Cell lysis and immunoblotting

Cultured cells were lysed in Pierce IP Lysis Buffer supplemented with cOmplete ULTRA Protease Inhibitor Cocktail Tablets (Roche).

Antibodies for immunoblots were GAPDH (D16H11, Cell Signaling), NOS1 (sc-5302, Santa Cruz Biotechnology), NOS2 (18985-1-AP, Proteintech), and NOS3 (D9A5L, Cell Signaling).

### SNO-RAC

SNO-proteomes were assessed by SNO-RAC assay as previously described (62). Gels of SNO-RAC samples were stained with SilverQuest Silver Staining Kit (Invitrogen). Input lysates for SNO-RAC were stained with Coomassie Imperial Protein Stain (ThermoFisher Scientific).

### Nitrite assay

Nitrite in cell culture medium was measured by Griess assay as previously described (11) using 1% sulfanilamide in 0.5 N HCl and N-(1-napthyl)ethylenediamine. Absorbance at 540 nm was compared against a sodium nitrite standard curve.

## Conflict of interest

The authors declare the following financial interests/personal relationships, which may be considered as potential competing interests: Jonathan Stamler is a founder and board member of and has equity interest in SNO bio, a company developing nitrosylation-related therapeutics, and NNOXX, a company developing NO-based device technology. CWRU and UHCMC are aware of these conflicts, and appropriate management plans are in place. None of the other authors has relevant conflicts to disclose.
